# Steep medial posterior tibial slope angle and ramp lesion are independent risk factors for an increase in quantitatively measured pivot shift in patients with ACL‐deficient knees

**DOI:** 10.1002/jeo2.70011

**Published:** 2024-08-28

**Authors:** Ryu Yoshida, Hideyuki Koga, Tomomasa Nakamura, Yusuke Nakagawa, Mai Katakura, Masaki Amemiya, Takashi Hoshino, Aritoshi Yoshihara, Shoichi Hasegawa, Yasumasa Tokumoto, Ichiro Sekiya, Nobutake Ozeki

**Affiliations:** ^1^ Department of Joint Surgery and Sports Medicine Tokyo Medical and Dental University Bunkyo‐ku, Tokyo Japan; ^2^ Department of Cartilage Regeneration Tokyo Medical and Dental University Tokyo Japan; ^3^ Center for Stem Cell and Regenerative Medicine Tokyo Medical and Dental University Tokyo Japan

**Keywords:** anterior cruciate ligament reconstruction, knee rotational instability, pivot shift, risk factors, triaxial accelerometer

## Abstract

**Background:**

Delayed anterior cruciate ligament (ACL) reconstruction often causes residual anterolateral rotatory instability (ALRI) and consequent knee osteoarthritis, warranting the recommendation of early ACL reconstruction within 6 months after injury. Nonetheless, some cases show notable instability, even shortly after injury. The purpose of this study was to identify risk factors for an increase in quantitatively measured pivot shift in patients with ACL‐deficient knees within 6 months after injury.

**Methods:**

Patients with primary ACL reconstruction within 6 months after injury and quantitative triaxial accelerometer measurements of preoperative pivot shift were included. Descriptive statistics were calculated for 11 independent variables (age, gender, time from injury to surgery, KT‐1000, knee extension angle, lateral and medial posterior tibial slope angle, medial and lateral meniscus tears, ramp lesion and Tegner active scale). A single regression analysis was performed on the 11 items and acceleration during the pivot shift, and a multiple regression analysis was performed for items with *p* value less than 0.1.

**Results:**

Overall, 111 patients met the inclusion criteria. Single regression analysis showed that medial posterior tibial slope angle, medial meniscus tear and ramp lesion were significantly correlated with acceleration during the pivot shift (*p* < 0.001, *p* = 0.04 and *p* = 0.001). Multiple regression analysis identified medial posterior tibial slope angles and ramp lesions as independent factors for increased preoperative pivot shift (*p* = 0.005 and 0.01).

**Conclusion:**

A steep medial posterior tibial slope angle and ramp lesion were independent risk factors for increased quantitatively measured pivot shift in patients with ACL‐deficient knees.

**Level of Evidence:**

Level IV, case series study.

**Abbreviations**
ACL: anterior cruciate ligament
ALRI: anterolateral rotatory instability
ICC: intraclass correlation coefficient
IKDC: International Knee Documentation Committee
KiRA: kinematic rapid assessment
PCL: posterior cruciate ligament

## INTRODUCTION

Residual anterolateral rotatory instability (ALRI) persisting after anterior cruciate ligament (ACL) reconstruction, as evaluated through the pivot shift test, is a risk factor for knee osteoarthritis [8] and correlates with reduced patient satisfaction [11]. ACL reconstruction is recommended within 6 months following injury, as prolonged periods between injury and reconstruction may lead to further damage to the meniscus and articular cartilage, potentially exacerbating ALRI [17]. Nevertheless, even when ACL reconstruction surgery is performed within 6 months postinjury, some cases may still show significant postoperative ALRI.

A high degree of preoperative pivot shift has been reported to increase the risk of residual pivot shift after ACL reconstruction [9, 27]. Therefore, proper evaluation of the pivot shift phenomenon should be conducted both before and after ACL reconstruction. The assessment of the pivot shift phenomenon has traditionally relied on subjective methods [1, 13, 14, 19]; however, the recent development of various measurement techniques utilizing electromagnetic waves and accelerometers has enabled quantitative assessment of the pivot shift phenomenon in ACL‐injured knees [2, 12, 15], with the aim of providing an objective assessment of ALRI.

The kinematic rapid assessment (KiRA) triaxial accelerometer (OrthoKey) is a device capable of measuring tibial acceleration during the pivot shift [14, 18]. KiRA measurements in ACL‐reconstructed knees have revealed a significant decrease in acceleration of the pivot shift compared to the preoperative level [18]. However, no analysis has yet been conducted using the triaxial accelerometer to investigate the risk factors associated with the increased preoperative quantitative pivot shift phenomenon.

The aim of this study was to use the triaxial accelerometer to investigate the factors influencing the quantitative pivot shift phenomenon in ACL‐injured knees within 6 months of injury, with the goal of identifying cases with a high risk of pivot shift even shortly after injury. Our hypothesis was that concomitant meniscal injury and tibial plateau anatomy would increase the risk of preoperative ALRI as assessed using the triaxial accelerometer.

## PATIENTS AND METHODS

### Patients

This retrospective study was approved by the Institutional Review Board at Tokyo Medical and Dental University (research protocol identification number: M2000‐1566). Between June 2014 and August 2023, a consecutive series of patients who had undergone primary double‐bundle ACL reconstruction with an autologous semitendinosus tendon within 6 months after injury were included in this study. Nine surgeons in the same group operated on the patients with the same method. There are no age restrictions on ACLR. Patients with previous knee trauma or compound ligament injuries (with concomitant injuries of posterior cruciate ligament (PCL), medial collateral ligament and/or lateral collateral ligament), as well as patients with contralateral injuries, were excluded. Cases in which meniscus procedures were performed together with ACLR were included in the study, while osteotomy and cartilage surgery were excluded. Patients were certainly taken the plain radiograph of bilateral knees and magnetic resonance imaging just before surgeries.

### Data collection

The demographic and image‐measurement data were collected using the following methods. Data on (1) age (1‐year increments), (2) gender (male or female) and (3) time from injury to surgery (0.5‐month increments) were obtained during the initial interview. Preoperative anterior knee laxity was measured in 0.5 mm increments under anaesthesia in both injured and uninjured knees by five knee surgeons with more than 15 years of experience in the same group using a (4) KT‐1000 arthrometer (MEDmetric) [27]. The differences in laxity between the injured and uninjured knees were scored. (5) The knee extension angle was measured in 1° increments using the preoperative lateral view of the plain radiograph of the uninjured knee [27]. (6) The lateral posterior tibial slope angle and (7) medial posterior tibial slope angle were measured with MR images in 0.1° increments according to the three‐step method [6]. The central sagittal slice was first defined by the intercondylar eminence and then by the anterior and posterior tibial cortices that appeared concave. The tibial attachment of the PCL was defined and determined as the tibial longitudinal axis, and the angle between a line perpendicular to the tibial longitudinal axis and the tangent lines to the medial and lateral tibial plateaus was measured. Angle measurements were taken by an orthopaedic doctor (R. Y.). At least one month later, angles were measured again by the same assessor in 10 random cases, whereby the intraclass correlation coefficient (ICC) was measured. The intra‐ICC of the angle measurements was 0.97. (8) Medial meniscus tears, especially (9) ramp lesions and (10) lateral meniscus tears were diagnosed by senior knee surgeons using arthroscopy. (11) The Tegner activity scale was used as an indicator of the activity level prior to injury; this is a measure of specific exercise activity and is given a score of 10 for athletes who exercise at the competitive level and 0 for those who have no exercise habits at the daily living level [26].

### Evaluation of ALRI

**Figure 1:**
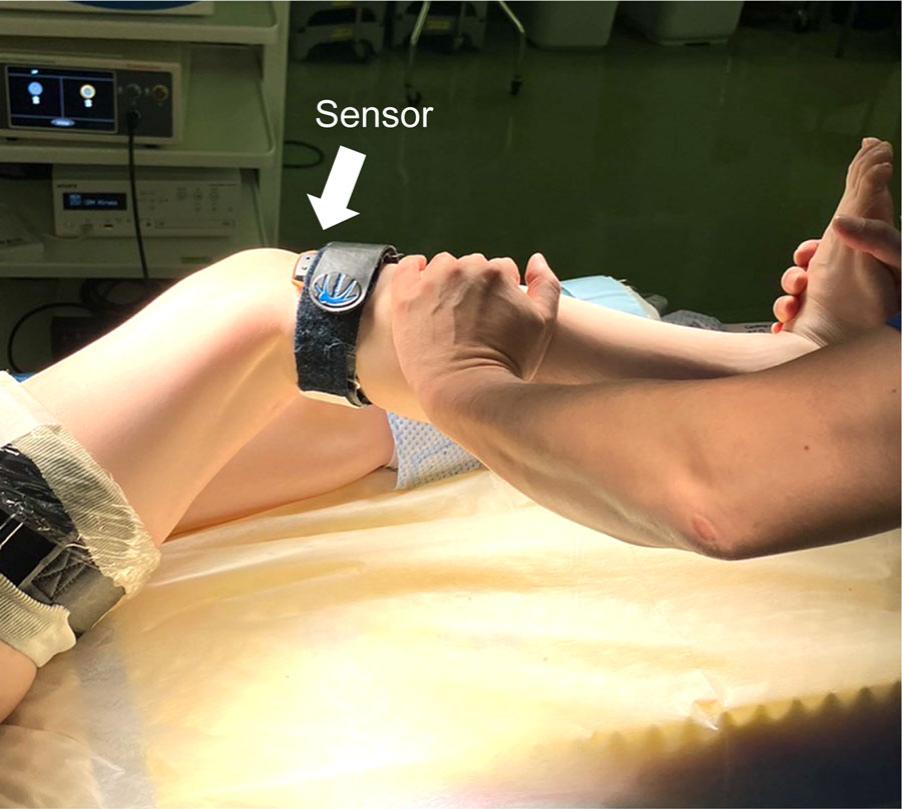
Evaluation of the pivot shift with a triaxial accelerometer. White arrow: a sensor (placed between the lateral aspect of the tibia and a band).

**Figure 2:**
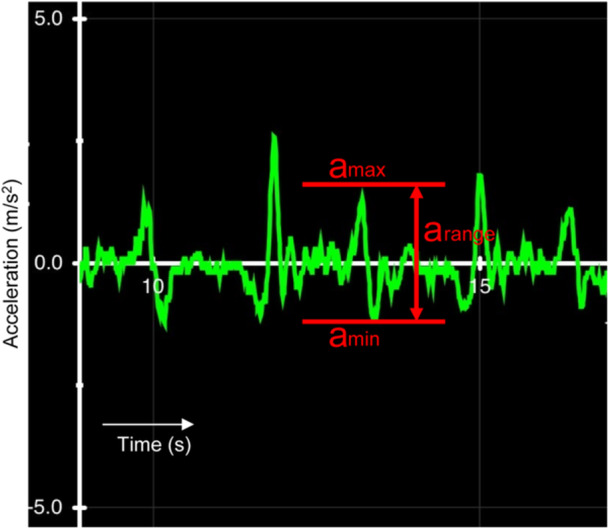
A representative wave during the pivot‐shift test obtained by KiRA. The difference between the maximum value (*a*_max_) and minimum value (*a*_min_) of the acceleration was calculated (*a*_range_ = *a*_max_ − *a*_min_).

(A) Pivot shift based on seven‐grade subjective evaluation
  ALRI was assessed using a pivot shift test performed with the patients under anaesthesia. Two experienced orthopaedic surgeons each performed an assessment before surgery and reached a consensus on the grade in every case. In the test, the modified International Knee Documentation Committee (IKDC) criteria [12] (Grade 0 = negative; Grade 1 = subtle glide, but not negative; Grade 2 = glide; Grade 3 = between Grades 2 and 4; Grade 4 = clunk; Grade 5 = between Grades 4 and 6; Grade 6 = gross) were used as the grading system to evaluate the pivot shift phenomenon. A previous report using an analysis of interobserver reliability showed that the inter‐ICC for the seven‐grade pivot shift test was 0.778 (95% confidence interval: 0.437–0.912) [18].
(B) Pivot shift based on the triaxial accelerometer
  Quantitative assessment of the pivot shift phenomenon was performed by the same surgeons before surgery using the KiRA triaxial accelerometer (Figure 1). The intra‐ICC of the KiRA is reported as 0.88–0.89 [21]. A built‐in three‐axis acceleration sensor was attached to the proximal lateral tibia, and the movement of the tibia during the pivot shift test was analysed through a wireless signal connected to a tablet terminal. The difference (range = *a*_max_ − *a*_min_) between the maximum (*a*_max_) and minimum (*a*_min_) values of acceleration (m/s^2^) in the pivot shift test measured by triaxial acceleration was evaluated as the size of subluxation (Figure 2). Each pivot shift test was conducted five times, and the acceleration was measured for every test. Maximum and minimum values were excluded, and the three remaining data were averaged and used for the analysis.

### Data analysis

The descriptive statistics, such as mean, standard deviation and minimum–maximum values, were first calculated for the 10 independent variables (age, gender, time from injury to surgery, KT‐1000, knee extension angle, lateral and medial posterior tibial slope angle, medial and lateral meniscus tear and medial meniscus ramp lesion) and the Tegner active scale score was expressed as median and minimum–maximum values. A single regression analysis was then performed on these 11 demographic and clinical data items as independent variables using the pivot shift phenomenon (triaxial accelerometer readings) as a dependent variable. A multivariate linear regression analysis was then performed on variables for which the *p* value was less than 0.1.

The sample size calculation was performed using G*Power (version 3.1.9.6) [5]. Assuming a multiple regression model with four explanatory variables for which we want to estimate the partial regression coefficients, and given an effect size of *f* ^2^ = 0.15, when testing at a significance level of 5% and a power of 80%, the required sample size was calculated as a total of 85 cases. The data were analysed using Prism (version 9.5.0; GraphPad Software, LLC). In all analyses, a *p* value less than 0.05 was considered statistically significant.

## RESULTS

### Patient characteristics

Out of the 674 patients, data were analysed for 111 patients who met the inclusion criteria by undergoing surgery within 6 months of injury, excluding those with complex ligament injuries or revisions. The mean age was 25 ± 9 years (range: 13–50), with 60 males and 51 females. The mean time to surgery was 2.7 ± 1.5 months (range: 0.5–6.0). The KT‐1000 showed differences between injured and uninjured knees of 6.1 ± 2.2 mm (range: 1.0–13.0), and the knee extension angle was 6 ± 4° (range: −9 to 16). The lateral posterior tibial slope angle was 5.8 ± 3.2° (range: 0.1–13.4), and the medial posterior tibial slope angle was 5.6 ± 2.8° (range 0.1–13.0). The cases included 60 resections or repair of a medial meniscus (MM) tear (especially 18 cases of MM ramp lesions) and 66 cases of lateral meniscus tears during ACL reconstruction. The median Tegner activity scale score was 7 (range: 3–9) (Table 1). The pivot shift test grades, evaluated on a seven‐grade scale, are shown in Table 2. The pivot shift phenomenon, based on the triaxial accelerometer (m/s^2^), had an average acceleration of 5.9 ± 3.2 m/s^2^ (range: 0.9–19.9) (Table 1).

**Table 1:** Patient's data.

		Unit	Mean ± SD or median	Range
Age		Years old	25 ± 9	13–50
Gender	Male 60, female 51			
Time to surgery		Months	2.7 ± 1.5	0–6
KT‐1000 difference		mm	6.1 ± 2.2	1.0–13
Knee extension angle		Degree	6 ± 4	−9 to 16
Lateral posterior tibial slope angle		Degree	5.8 ± 3.2	0.1–13.4
Medial posterior tibial slope angle		Degree	5.6 ± 2.8	0.1–13
MM tear	60 cases (ramp 18 cases)			
LM tear	66 cases			
Tegner activity score			7	3–9
Pivot shift based on triaxial accelerometer		m/s^2^	5.9 ± 3.2	0.9–19.9

Abbreviations: LM, lateral meniscus; MM, medial meniscus; SD, standard deviation.

**Table 2:** Pivot shift based on seventh‐grade evaluation.

	Grade 0	Grade 1	Grade 2	Grade 3	Grade 4	Grade 5	Grade 6	All
*n*	0	4	3	28	54	21	1	111

### Correlation of the subjective and quantitative pivot shift phenomenon

**Figure 3:**
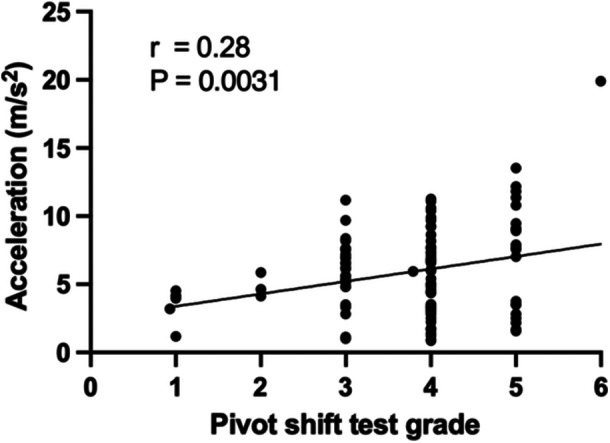
Evaluation under anaesthesia. Correlations between the pivot shift test subjective grading and acceleration in anterior cruciate ligament‐injured knees during the pivot shift test.

The pivot shift test subjective grade and acceleration measured with the triaxial accelerometer during the pivot shift under anaesthesia showed a weak positive correlation, with a correlation coefficient of 0.28 (*p* = 0.003) (Figure 3).

### Factors associated with the preoperative quantitatively assessed pivot shift

A single regression analysis revealed that the preoperative pivot shift acceleration was significantly correlated with the medial posterior tibial slope angle (*r* = 0.257, *p* < 0.001), MM tear (*r* = 0.191, *p* = 0.044) and ramp lesion (*r* = 0.303, *p* = 0.001) (Table 3). Further multiple regression analysis revealed that the medial posterior tibial slope angle (*β* = 0.265, *p* = 0.005) and ramp lesion (*β* = 2.17, *p* = 0.010) were significant independent factors for an increase in pivot shift acceleration, as measured using the triaxial accelerometer (Table 4).

**Table 3:** Univariate analysis of candidate risk factors.

		Absolute value of acceleration	
Explanatory variable	Unit	*p*	*r*
Age	Years	0.381	0.084
Gender		0.063	0.177
Time to surgery	Months	0.598	0.051
KT‐1000 difference^a^		0.230	0.115
Knee extension angle	Degrees	0.820	0.022
Lateral posterior tibial slope angle	Degrees	0.187	0.126
Medial posterior tibial slope angle	Degrees	<0.001	0.257
MM tear		0.044	0.191
MM ramp lesion		0.001	0.303
LM tear		0.114	0.151
Tegner activity score		0.629	0.046

Abbreviations: LM, lateral meniscus; MM, medial meniscus.

a Difference between anterior cruciate ligament‐injured side and intact side.

**Table 4:** Multivariate analysis of candidate risk factors.

Variable	*β*	*p*
Absolute value of acceleration (*R* ^2^ = 0.19)		
Gender	0.915	0.110
Medial posterior tibial slope angle	0.265	0.005
MM tear	0.870	0.166
MM ramp lesion	2.17	0.010

Abbreviations: MM, medial meniscus; *R* ^2^, adjusted *R* ^2^; *β*, standardized beta coefficients.

## DISCUSSION

The most important finding of this study was that a steep medial posterior tibial slope angle and the presence of ramp lesion were risk factors associated with increased pivot shift phenomenon in ACL‐deficient knees within 6 months after injury, as assessed using the triaxial accelerometer. Furthermore, only a weak correlation was observed between the acceleration measured by the triaxial accelerometer and the subjective grade of the pivot shift phenomenon, indicating that a more quantitative measurement of pivot shift provided a more precise assessment of the risk factors leading to an increased pivot shift.

A significant association has previously been reported between ALRI and MM ramp lesions in ACL‐deficient knees [3, 25]. A cadaveric study demonstrated that ramp lesions increased forces on the ACL and that lesions of the meniscotibial ligament increased rotational instability of the knee joint [20]. A biomechanical study revealed that ramp lesions in ACL‐injured knees induced a pivot shift phenomenon and that repairing these lesions during ACL reconstruction could effectively reduce knee instability [3]. In the present study, the multivariate analysis identified ramp lesions as an independent risk factor for greater triaxial acceleration, suggesting that ramp lesions be treated during ACL reconstruction, as recommended in previous reports [22].

The findings of the present study also identified a steep medial posterior tibial slope angle as an independent risk factor for increasing the pivot shift phenomenon. The posterior tibial slope angle has an association with the stability of the knee joint [4, 23], as an increase in the posterior tibial tilt angle has a clear association between knee joint instability and increased loading on the MM, particularly at the posterior root of the MM [23]. Increases in the medial posterior tibial tilt angle are associated with a higher incidence of ramp lesions associated with ACL tears [7, 10]. Several reports have suggested that reducing the posterior slope of the tibia is effective in improving instability in ACL‐deficient knees [16, 28]. Proper preoperative evaluation of the pivot shift phenomenon is therefore crucial in knees with a significant posterior slope for optimal management

Several risk factors for a large pivot shift phenomenon have been reported using a manual pivot shift test. For example, Kamada et al. performed a manual pivot shift test under anaesthesia during implant removal after double‐bundle ACLR and reported that age at surgery <20 years, preoperative high‐grade pivot shift, and hyperextended knee were high‐risk factors for residual postoperative pivot shift [9]. Song et al. performed a manual pivot shift test on 90 knees with ACL reconstruction within 3 weeks of injury and identified pivoting sports at the time of injury, increased posterior‐inferior tibial slope, anterolateral capsular ligament disruptions and combined lateral meniscal lesions as preoperative risk factors that influenced the postoperative persistence of a large pivot shift phenomenon [24]. In these reports, the pivot shift tests were performed manually, with the poor reproducibility of the results cited as a limitation. By contrast, in the current study, the quantitative measurements of the pivot shift using the highly reproducible triaxial accelerometer had an ICC of 0.88–0.89. Therefore, the current identification of an independent risk associated with a steep medial posterior tibial slope angle and ramp lesion for a large preoperative pivot shift can lead to more reliable outcomes.

The main limitation of this study is that it was conducted retrospectively. Furthermore, the pivot shift tests were not performed blindly, and the technique was not necessarily consistent, as it was conducted by multiple examiners. However, efforts were made to ensure consistency in technique by attempting a more rigorous evaluation of pivot shift using the modified IKDC criteria. Additionally, to minimize variability among evaluators, each test was performed five times, with the highest and lowest values excluded and the remaining three values averaged. Finally, the present study did not consider detailed information on meniscus injuries and their treatment. The results of this study did not show that anything related to the meniscus was a risk, but it could have been the risk by analysing the detailed meniscus injury type.

The clinical relevance of this study is that risk factors for ALRI within 6 months of ACL injury were obtained using a triaxial accelerometer. The usage of a triaxial accelerometer for a more reproducible pivot‐shift assessment might help surgeons to better identify potential adverse biomechanical consequences of MM ramp lesions and increased tibial slope. This could lead to performing additional anterolateral structure augmentation and/or anterior closing‐wedge osteotomy in combination with ACL reconstruction.

In conclusion, steep medial posterior tibial slope angles and ramp lesions were independent risk factors for increases in quantitatively measured pivot shifts in patients with ACL‐deficient knees.

## AUTHOR CONTRIBUTIONS

Ryu Yoshida managed data, performed statistical analysis, participated in study design and wrote the manuscript. Hideyuki Koga contributed to the acquisition of data and participated in the study design. Tomomasa Nakamura, Yusuke Nakagawa, Mai Katakura, Masaki Amemiya, Takashi Hoshino, Aritoshi Yoshihara, Shoichi Hasegawa, Yasumasa Tokumoto and Ichiro Sekiya acquisition of data. Nobutake Ozeki contributed to the acquisition of data, participated in study design, interpreted results, edited the manuscript, and had full access to all of the data in the study and final approved manuscript. All authors read, approved the final manuscript and take responsibility for the integrity of the data and the accuracy of the data analysis.

## CONFLICT OF INTEREST STATEMENT

The authors declare no conflict of interest.

## ETHICS STATEMENT

This study was approved by the Institutional Review Board at Tokyo Medical and Dental University (research protocol identification number: M2000‐1566). All study participants provided their full written informed consent for participation in this clinical research prior to the operative procedure.

## Data Availability

The data sets generated and/or analysed during the current study are not publicly available due to a lot of personally identifiable information but are available from the corresponding author on reasonable request.

## References

[1] Benjaminse, A. , Gokeler, A. & van der Schans, C.P. (2006) Clinical diagnosis of an anterior cruciate ligament rupture: a meta‐analysis. Journal of Orthopaedic & Sports Physical Therapy, 36, 267–288. Available from: 10.2519/jospt.2006.2011 16715828

[2] Borgstrom, P.H. , Markolf, K.L. , Foster, B. , Petrigliano, F.A. & McAllister, D.R. (2014) Use of a gyroscope sensor to quantify tibial motions during a pivot shift test. Knee Surgery, Sports Traumatology, Arthroscopy, 22, 2064–2069. Available from: 10.1007/s00167-013-2610-0 23884298

[3] DePhillipo, N.N. , Moatshe, G. , Brady, A. , Chahla, J. , Aman, Z.S. , Dornan, G.J. et al. (2018) Effect of meniscocapsular and meniscotibial lesions in ACL‐deficient and ACL‐reconstructed knees: a biomechanical study. The American Journal of Sports Medicine, 46, 2422–2431. Available from: 10.1177/0363546518774315 29847148

[4] Familiari, F. , Tollefson, L.V. , Izzo, A. , Mercurio, M. , LaPrade, R.F. & Di Vico, G. (2024) A high‐grade Lachman's exam predicts a ramp tear of the medial meniscus in patients with anterior cruciate ligament tear: a prospective clinical and radiological evaluation. Journal of Clinical Medicine, 13, 683. Available from: 10.3390/jcm13030683 38337378 PMC10856171

[5] Faul, F. , Erdfelder, E. , Lang, A.G. & Buchner, A. (2007) G*Power 3: a flexible statistical power analysis program for the social, behavioral, and biomedical sciences. Behavior Research Methods, 39, 175–191. Available from: 10.3758/BF03193146 17695343

[6] Hudek, R. , Schmutz, S. , Regenfelder, F. , Fuchs, B. & Koch, P.P. (2009) Novel measurement technique of the tibial slope on conventional MRI. Clinical Orthopaedics & Related Research, 467, 2066–2072. Available from: 10.1007/s11999-009-0711-3 19190973 PMC2706341

[7] Jiang, J. , Liu, Z. , Wang, X. , Xia, Y. & Wu, M. (2022) Increased posterior tibial slope and meniscal slope could be risk factors for meniscal injuries: a systematic review. Arthroscopy: The Journal of Arthroscopic & Related Surgery, 38, 2331–2341. Available from: 10.1016/j.arthro.2022.01.013 35066109

[8] Jonsson, H. , Riklund‐Åhlström, K. & Lind, J. (2004) Positive pivot shift after ACL reconstruction predicts later osteoarthrosis: 63 patients followed 5‐9 years after surgery. Acta Orthopaedica Scandinavica, 75, 594–599. Available from: 10.1080/00016470410001484 15513493

[9] Kamada, K. , Matsushita, T. , Nagai, K. , Hoshino, Y. , Araki, D. , Kanzaki, N. et al. (2023) Risk factors of residual pivot‐shift after anatomic double‐bundle anterior cruciate ligament reconstruction. Archives of Orthopaedic and Trauma Surgery, 143, 977–985. Available from: 10.1007/s00402-022-04428-y 35364734

[10] Kim, S.H. , Seo, H.J. , Seo, D.W. , Kim, K.I. & Lee, S.H. (2020) Analysis of risk factors for ramp lesions associated with anterior cruciate ligament injury. The American Journal of Sports Medicine, 48, 1673–1681. Available from: 10.1177/0363546520918207 32383965

[11] Kocher, M.S. , Steadman, J.R. , Briggs, K.K. , Sterett, W.I. & Hawkins, R.J. (2004) Relationships between objective assessment of ligament stability and subjective assessment of symptoms and function after anterior cruciate ligament reconstruction. The American Journal of Sports Medicine, 32, 629–634. Available from: 10.1177/0363546503261722 15090377

[12] Kuroda, R. , Hoshino, Y. , Araki, D. , Nishizawa, Y. , Nagamune, K. , Matsumoto, T. et al. (2012) Quantitative measurement of the pivot shift, reliability, and clinical applications. Knee Surgery, Sports Traumatology, Arthroscopy, 20, 686–691. Available from: 10.1007/s00167-011-1849-6 22210517

[13] Kuroda, R. , Hoshino, Y. , Kubo, S. , Araki, D. , Oka, S. , Nagamune, K. et al. (2012) Similarities and differences of diagnostic manual tests for anterior cruciate ligament insufficiency: a global survey and kinematics assessment. The American Journal of Sports Medicine, 40, 91–99. Available from: 10.1177/0363546511423634 21989128

[14] Lopomo, N. , Zaffagnini, S. & Amis, A.A. (2013) Quantifying the pivot shift test: a systematic review. Knee Surgery, Sports Traumatology, Arthroscopy, 21, 767–783. Available from: 10.1007/s00167-013-2435-x 23455384

[15] Lopomo, N. , Zaffagnini, S. , Bignozzi, S. , Visani, A. & Marcacci, M. (2010) Pivot‐shift test: analysis and quantification of knee laxity parameters using a navigation system. Journal of Orthopaedic Research, 28, 164–169. Available from: 10.1002/jor.20966 19642114

[16] Mabrouk, A. , Kley, K. , Jacquet, C. , Fayard, J.M. , An, J.S. & Ollivier, M. (2023) Outcomes of slope‐reducing proximal tibial osteotomy combined with a third anterior cruciate ligament reconstruction procedure with a focus on return to impact sports. The American Journal of Sports Medicine, 51, 3454–3463. Available from: 10.1177/03635465231203016 37885232

[17] Nakamae, A. , Miyamoto, A. , Kamei, G. , Eguchi, A. , Shimizu, R. , Akao, M. et al. (2022) An older age, a longer duration between injury and surgery, and positive pivot shift test results increase the prevalence of articular cartilage injury during ACL reconstruction in all three compartments of the knee in patients with ACL injuries. Knee Surgery, Sports Traumatology, Arthroscopy, 30, 219–230. Available from: 10.1007/s00167-021-06461-7 33543356

[18] Nakamura, K. , Koga, H. , Sekiya, I. , Watanabe, T. , Mochizuki, T. , Horie, M. et al. (2017) Evaluation of pivot shift phenomenon while awake and under anaesthesia by different manoeuvres using triaxial accelerometer. Knee Surgery, Sports Traumatology, Arthroscopy, 25, 2377–2383. Available from: 10.1007/s00167-015-3740-3 26233597

[19] Noyes, F.R. , Grood, E.S. , Cummings, J.F. & Wroble, R.R. (1991) An analysis of the pivot shift phenomenon. The knee motions and subluxations induced by different examiners. The American Journal of Sports Medicine, 19, 148–155. Available from: 10.1177/036354659101900210 2039066

[20] Peltier, A. , Lording, T. , Maubisson, L. , Ballis, R. , Neyret, P. & Lustig, S. (2015) The role of the meniscotibial ligament in posteromedial rotational knee stability. Knee Surgery, Sports Traumatology, Arthroscopy, 23, 2967–2973. Available from: 10.1007/s00167-015-3751-0 26264383

[21] Raggi, F. , Roberti di Sarsina, T. , Signorelli, C. , Marcheggiani Muccioli, G.M. , Macchiarola, L. , Cucurnia, I. et al. (2019) Triaxial accelerometer can quantify the Lachman test similarly to standard arthrometers. Knee Surgery, Sports Traumatology, Arthroscopy, 27, 2698–2703. Available from: 10.1007/s00167-018-5306-7 30474693

[22] Rodriguez, A.N. , LaPrade, R.F. & Geeslin, A.G. (2022) Combined meniscus repair and anterior cruciate ligament reconstruction. Arthroscopy: The Journal of Arthroscopic & Related Surgery, 38, 670–672. Available from: 10.1016/j.arthro.2022.01.003 35248223

[23] Shu, L. , Abe, N. , Li, S. & Sugita, N. (2022) Importance of posterior tibial slope in joint kinematics with an anterior cruciate ligament‐deficient knee. Bone & Joint Research, 11, 739–750. Available from: 10.1302/2046-3758.1110.BJR-2022-0039.R1 36226477 PMC9582864

[24] Song, G. , Zhang, H. , Wang, Q. , Zhang, J. , Li, Y. & Feng, H. (2016) Risk factors associated with grade 3 pivot shift after acute anterior cruciate ligament injuries. The American Journal of Sports Medicine, 44, 362–369. Available from: 10.1177/0363546515613069 26620298

[25] Stephen, J.M. , Halewood, C. , Kittl, C. , Bollen, S.R. , Williams, A. & Amis, A.A. (2016) Posteromedial meniscocapsular lesions increase tibiofemoral joint laxity with anterior cruciate ligament deficiency, and their repair reduces laxity. The American Journal of Sports Medicine, 44, 400–408. Available from: 10.1177/0363546515617454 26657852

[26] Tegner, Y. & Lysholm, J. (1985) Rating systems in the evaluation of knee ligament injuries. Clinical Orthopaedics and Related Research, 198, 43–49. 4028566

[27] Ueki, H. , Nakagawa, Y. , Ohara, T. , Watanabe, T. , Horie, M. , Katagiri, H. et al. (2018) Risk factors for residual pivot shift after anterior cruciate ligament reconstruction: data from the MAKS group. Knee Surgery, Sports Traumatology, Arthroscopy, 26, 3724–3730. Available from: 10.1007/s00167-018-5005-4 29947841

[28] Weiler, A. , Berndt, R. , Wagner, M. , Scheffler, S. , Schatka, I. & Gwinner, C. (2023) Tibial slope on conventional lateral radiographs in anterior cruciate ligament‐injured and intact knees: mean value and outliers. The American Journal of Sports Medicine, 51, 2285–2290. Available from: 10.1177/03635465231178292 37306059 PMC10353028

